# Discovering the ‘Dark matters’ in expression data of miRNA based on the miRNA-mRNA and miRNA-lncRNA networks

**DOI:** 10.1186/s12859-018-2410-0

**Published:** 2018-10-16

**Authors:** Cong Pian, Guangle Zhang, Sanling Wu, Fei Li

**Affiliations:** 1 Ministry of Agriculture Key Lab of Molecular Biology of Crop Pathogens and Insects, Institute of Insect Science, Zhejiang University, Hangzhou, 310058 Zhejiang China; 2 Department of Mathematics, College of Science, Nanjing Agricultural University, Nanjing, 210095 Jiangsu China; 3 Analysis Center of Agrobiology and Environmental Sciences, Zhejiang University, Hangzhou, 310058 Zhejiang China

**Keywords:** Dark matters, miRNA-mRNA network, miRNA-lncRNA network, Biomarkers

## Abstract

**Background:**

Since miRNAs can play important roles in different cancer types, how to discover cancer related miRNAs is an important issue. In general, the miRNAs with differential expression is the focus of attention. However, some important cancer related miRNAs are not excavated by differential expression analysis. We take this type of miRNAs as ‘dark matters’ (DM-miRNA). It is our great interests to develop an algorithm to discover DM-miRNAs.

**Results:**

An effective method was developed to find DM-miRNAs. This method is mainly for mining potential DM-miRNAs by building basic miRNA-mRNA network (BMMN) and miRNA-lncRNA network (BMLN). The results indicate that miRNA-mRNA and miRNA-lncRNA interactions can be used as novel cancer biomarkers.

**Conclusions:**

The BMMN and BMLN can excavate the non-differentially expressed miRNAs which play an important role in the cancer. What’s more, the edge biomarkers (miRNA-mRNA and miRNA-lncRNA interactions) contain more information than the node biomarkers. It will contribute to developing novel therapeutic candidates in cancers.

**Electronic supplementary material:**

The online version of this article (10.1186/s12859-018-2410-0) contains supplementary material, which is available to authorized users.

## Background

microRNAs (miRNAs) are small non-coding RNA with length of 20 ~ 24 nucleotides. In animals, they mainly inhibit target mRNAs translation by binding to the 3′ untranslated regions (3’ UTRs) of mRNA. miRNAs participate in a variety of physiological processes, such as cell proliferation and differentiation, apoptosis, embryonic development, virus defense, and the hematopoietic process [1, 2]. miRNAs have been implicated in many diseases, especially in various types of cancers, such as lung neoplasms [3], breast neoplasms [4], colonic neoplasms [5], etc. Identifying miRNAs that have essential roles in tumorigenesis is an important task. A direct method is to find differentially expressed miRNAs from expression data. Unfortunately, some non-differentially expressed miRNAs may also play important regulatory functions in cancer, whereas some differentially expressed miRNAs do not show obvious roles in cancer. Those miRNAs that are non-differentially expressed but with important roles in tumorigenesis seems like ‘dark matters’ in the expression data which is very difficult to be detected.

Complex diseases generally result from dysfunctions in regulatory networks rather than from the mutations or malfunctions of a single molecule. We reasoned that miRNA-mRNA interactions may provide more information for discovering DM-miRNAs. To this end, we intend to develop an algorithm to identify DM-miRNA in the context of miRNA-mRNA interactions. Long non-coding RNAs (lncRNA) account for a large proportion in transcriptome. Some act as a competing endogenous RNA (ceRNA) which play an important role in the initiation and progression of cancer [6, 7]. So, we also.

Generally, prior works of constructing miRNA-mRNA modules from expression data can be divided into two-step procedures. First, Student t-test is used to obtain differentially expressed mRNAs and miRNAs. In general, the important step of identification of potential miRNA biomarkers is screening differentially expressed miRNA. Liao, et al., used five miRNAs of 320 differentially expressed mRNAs for prognostic signature construction [8]. Li, et al., selected 26 differentially expressed mRNAs to construct regulatory pathways in prostate cancer [9]. Second, miRNAs-mRNA regulatory network is constructed by calculating the Pearson correlation coefficient between the miRNAs and mRNAs. For example, a regulatory network for colorectal cancer was built using limma algorithms to select differentially expressed miRNAs and mRNAs [10]. A causality discovery-based method was used to uncover causal regulatory relationship between miRNAs and mRNAs [11]. However, these methods have two weaknesses: (1) It is difficult to construct a regulatory network when the number of samples is limited. (2) Some important miRNAs are not excavated by differential expression analysis. Here, we reasoned that all samples shared a basic miRNA-mRNA network (BMMN) and a basic miRNA-lncRNA network (BMLN). The miRNAs significantly deviating from BMMN or BMLN are regarded as potential DM-miRNAs. The results indicated that this method is efficient in discovering DM-miRNAs. Moreover, our works showed that miRNA-mRNA and miRNA-lncRNA interactions could be used as effective cancer biomarkers.

## Methods

### Datasets

Six different cancer types were selected, including breast invasive carcinoma (BRCA), kidney renal clear cell carcinoma (KIRC), lung adenocarcinoma (LUAD), lung squamous cell carcinoma (LUSC), thyroid carcinoma (THCA), prostate adenocarcinoma (PRAD). The expression profiles of 1071 miRNAs, 12,727 lncRNAs and 20,530 mRNAs of six different cancer types were downloaded from The Cancer Genome Atlas (TCGA) database (http://cancergenome.nih.gov) (Table 1).

**Table 1:** The type and sample number of cancers

Cancer abbreviation	Full name of cancer	Number of cancer tissue sample	Number of paired normal tissue sample
BRCA	Breast invasive carcinoma	755	86
KIRC	Kidney renal clear cell carcinoma	255	71
THCA	Thyroid carcinoma	511	59
LUAD	Lung adenocarcinoma	445	19
LUSC	Lung squamous cell carcinoma	342	38
PRAD	Prostate adenocarcinoma	494	52

The 155,044 experimentally validated miRNA-mRNA interactions were obtained from the miRTarBase database [12]. In addition, we integrated experimentally validated and predicted miRNA-lncRNA interactions from starBase v2.0 [13], miRcode [14] and NPInter v3.0 [15]. After removing redundant associations, 155,653 miRNA-lncRNA interactions were obtained. The experimentally verified miRNA-disease associations were downloaded from HMDD v2.0 [16], which included experimentally-verified human miRNA and disease associations.

### The construction of BMMN and BMLN

The process of constructing BMMN in a cancer can be divided into the following three steps: (1) The reference network is built using the paired normal tissue samples. (2) To quantify the importance of miRNA-mRNA association, we constructed an individual specific miRNA-mRNA network (ISMMN) and individual specific miRNA-lncRNA network (ISMLN) for a cancer sample. Then the frequency of significant change in each miRNA-mRNA interaction was calculated in all cancer samples. (3) The BMMN and BMLN of this cancer can be constructed using above high frequency miRNA-mRNA interactions.

**Fig. 1:**
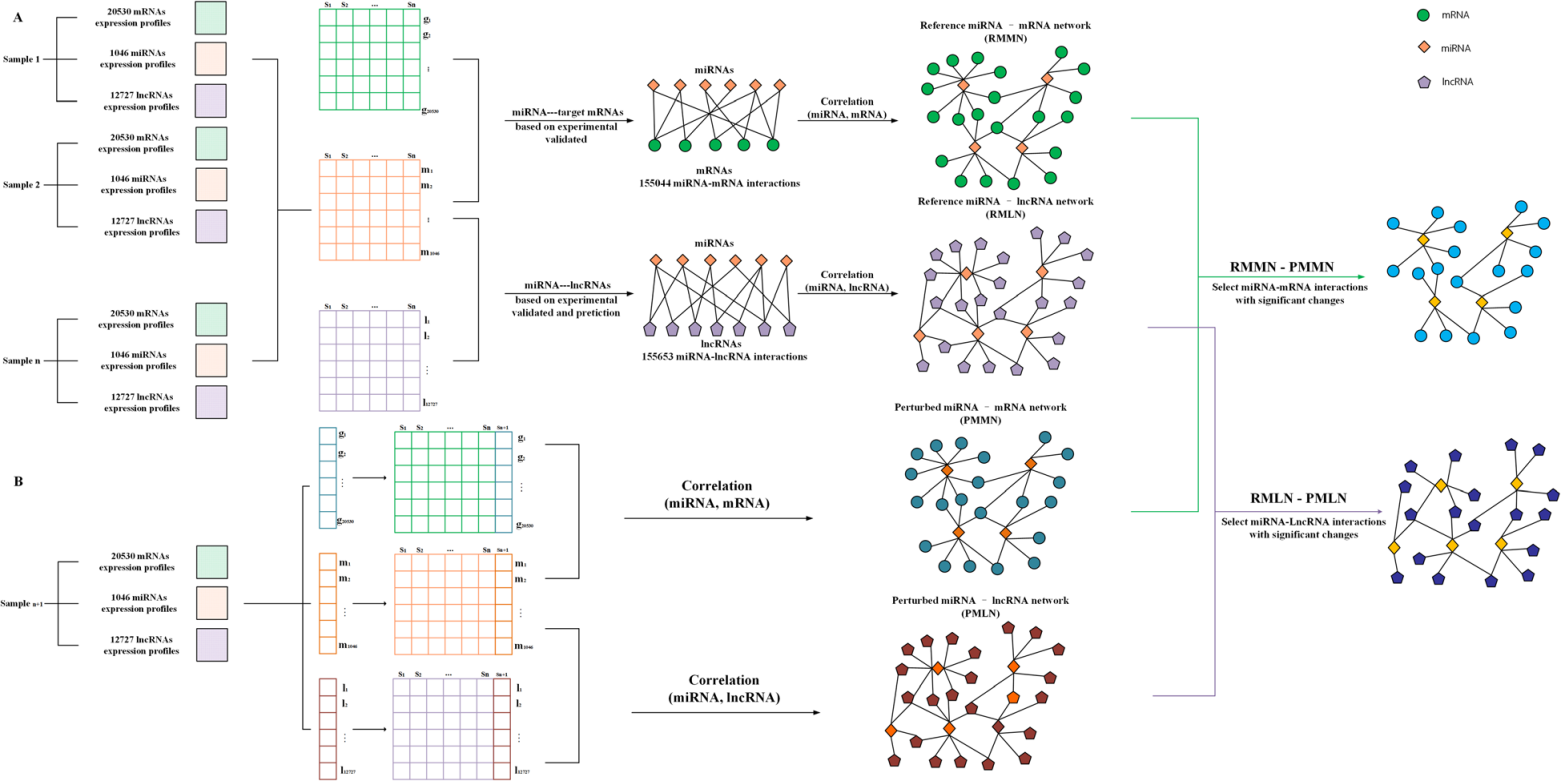
Flowchart for constructing an individual specific miRNA-mRNA network (ISMMN) and an individual specific miRNA-lncRNA network (ISMLN) for a single cancer sample. **a** The n normal samples are selected as the reference samples, and a reference miRNA-mRNA network (RMMN) and a reference miRNA-lncRNA network (RMLN) are constructed using Pearson Correlation Coefficients (PCC) based on the 155,044 miRNA-mRNA and 155,653 interactions. **b** The sample *S*_*n + 1*_ is added to the above reference samples, and a perturbed miRNA-mRNA network (PMMN) and a perturbed miRNA-mRNA network (PMLN) are built by recalculating the PPC. Finally, we define the difference (PMMN-RMMN) and (PMLN-RMLN) as the ISMMN and ISMLNof sample *S*_*n + 1*_

The processes for constructing ISMMN and ISMLN were given in Fig. 1. For each miRNA-mRNA pair, ${g}_1=\left({g}_1^1,{g}_1^2,\cdots, {g}_1^n\right),{m}_1=\left({m}_1^1,{m}_1^2,\cdots, {m}_1^n\right)$ represent the expression of mRNA and miRNA in n normal samples (*S*_1_, *S*_2_, ⋯, *S*_*n*_) We calculated Pearson correlation coefficients for each pair of miRNA-mRNA using g_1_ and m_1_ (${PCC}_{m_1,{g}_1}$) based on 155,044 experimentally verified human miRNA-mRNA interactions from the miRTarBase database. Finally, 155,044 Pearson correlation coefficients (*PCC*) were obtained, and a reference miRNA-mRNA regulatory network (RMMN) was generated (Fig. 1a). The reference network was generally stable with the increase of sample number. To build the ISMMN of the (*n + 1)*th sample S_n + 1_, we measured the disturbance degree of the reference miRNA-mRNA regulatory network by adding sample S_n + 1_ to the set (*S*_1_, *S*_2_, ⋯, *S*_*n*_).We then constructed the perturbed miRNA-mRNA regulatory network (PMMN) by calculating the PCC between ${g}_1^{\hbox{'}}=\left({g}_1^1,{g}_1^2,\cdots, {g}_1^n,{g}_1^{n+1}\right)$ and ${m}_1^{\hbox{'}}=\left({m}_1^1,{m}_1^2,\cdots, {m}_1^n,{m}_1^{n+1}\right)$ (${PCC}_{m_1^{\hbox{'}},{g}_1^{\hbox{'}}}$) (Fig. 1b). Finally, we calculated the differential miRNA-mRNA regulatory network between the reference regulatory network and perturbed regulatory network ∆*PCC=* $\left({PCC}_{m_1^1,{g}_1^{\hbox{'}}}-{PCC}_{m_1,{g}_1}\right)$*.* If the expression of the added sample were similar to that of reference samples, the fluctuation range of perturbed regulatory network would be insignificant. Finally, we select miRNA-mRNA interactions with high *∆PCC* value as the elements of ISMMN based on the statistical theory of Li et al. [17]. The procedures for constructing ISMLN was similar as that for ISMMN except lncRNAs were considered in instead of mRNA.

**Fig. 2:**
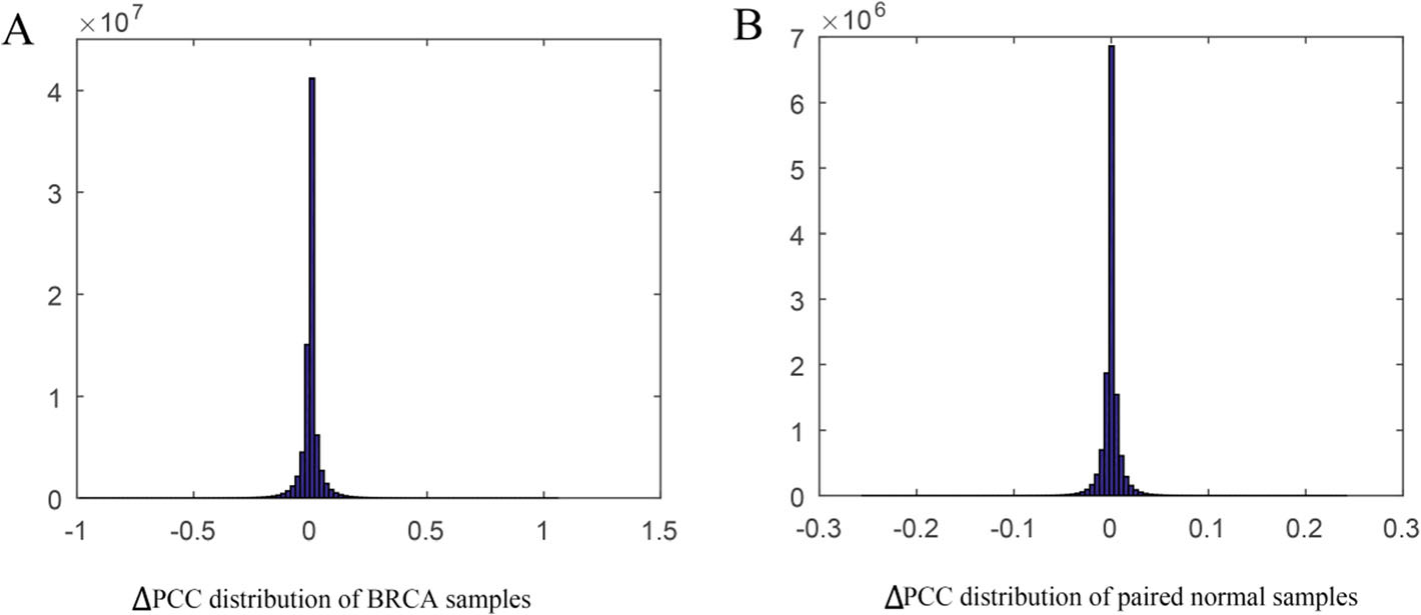
**a** and **b** represent the ∆PCC distributions of BRCA samples and paired normal samples. The X-axis represents the ∆*PCC* values in the BRCA samples and paired normal samples, respectively. The Y-axis represents the number of corresponding ∆*PCC* values. Most of ∆*PCCs* in normal samples tend to be 0 compared with it in BRCA samples. This result shows that there are obvious difference in miRNA-mRNA interactions between the cancer samples and normal samples

The distributions of *∆PCC* of BRCA samples and paired normal samples were given in Fig. 2. The X-axis and Y-axis represent the *∆PCC* value and corresponding frequency. There is a significant difference between the two distributions. The interval of X-axis are 0.5 and 0.1 in Fig. 2a and b. We can see that the ∆PCC values of BRCA samples are mainly concentrated on [− 0.5,0.5], while the region of ∆PCC of normal samples is [− 0.1,0.1]. The *P*-value of student T-test is 2.8705*10^− 156^. The results indicated that there were obviously changes in miRNA-mRNA interactions between cancer samples and normal samples, suggesting that cancer driving miRNA-mRNA interactions would cause significant changes in *PCC*. Therefore, we use all miRNA-mRNA interactions with significant changes to construct BMMN. The theory of previous study indicates that the significance level of ∆*PCC* can be evaluated by the statistical hypothesis test Z-test (or U-test) [17]. Assuming that there are n reference samples. Then we add the n + 1 sample, and the *PCC*_n_ and *PCC*_n + 1_ represent the edge of RMMN and PMMN. The ∆*PCC* of edge between RMMN and PMMN is ∆*PCC*_*n*_ *= PCC*_n + 1_ *-PCC*_n_. The significance of *∆PCC*_*n*_ can be evaluate by the following equation:

$$ Z=\frac{{\varDelta PCC}_n}{\frac{1- PCC{}_n{}^2}{n-1}} $$

**Fig. 3:**
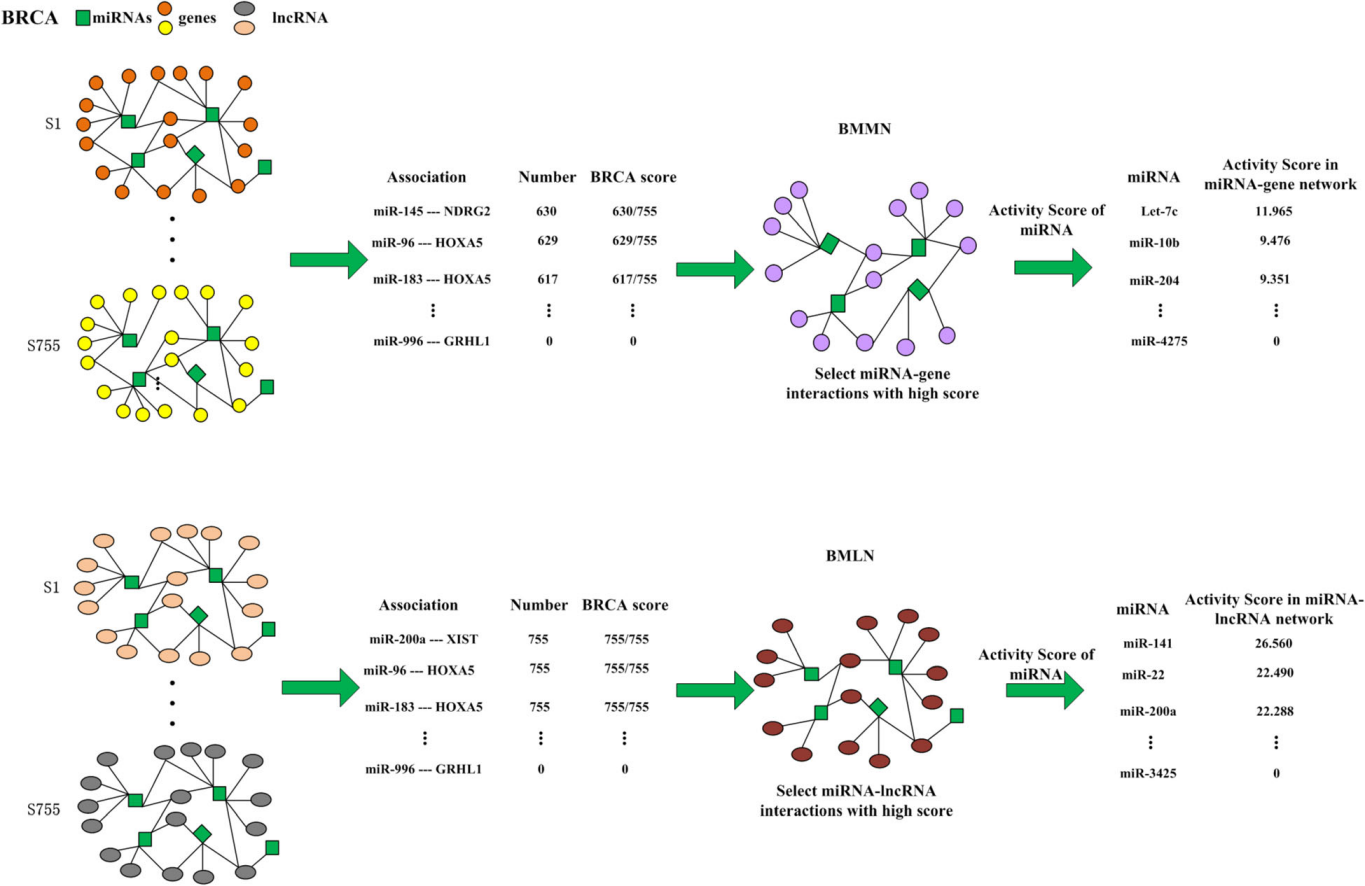
Construction of the BMMN in BRCA. The BRCA is used as the example. We built ISMMNs and ISMLN for the 755 breast cancer samples, counted the number of miRNA-mRNA and miRNA-lncRNA interactions that appeared in 755 breast ISMMNs and ISMLN, and ranked them in descending order. The BRCA score of every miRNA-mRNA and miRNA-lncRNA interaction can be calculated. Finally, the BMMN and BMLN of BRCA was constructed by selecting the miRNA-mRNA and miRNA-lncRNA interactions with high BRCA scores

ISMLN was built using the abovementioned method based on 155,653 miRNA-lncRNA interactions. BMMN and BMLN were constructed based on ISMMN and ISMLN. We used BRCA as the example (Fig. 3). First, we built ISMMN and ISMLN for 755 breast cancer samples. Second, we counted the number of miRNA-mRNA and miRNA-lncRNA interactions that appeared in 755 ISMMNs and ISMLNs. Then we ranked them in descending order. For example, the interaction of miR-145-NDRG2 changed significantly in 630 of 755 breast cancer samples. We set 0.834 (630/755) as the BRCA score of miR-145-NDRG2 interaction. The BRCA score of every miRNA-mRNA interaction were calculated. Finally, the BMMN and BMLN of BRCA were constructed by selecting miRNA-mRNA interactions with high BRCA scores. The construction of BMLN was similar to the process of BMMN. We set BRCA score = 0.4 as the threshold in the BMMN and BMLN.

### The calculation of miRNA activity scores in BMMN and BMLN

The formula of miRNA activity score in BMMN and BMLN are as follows:

$$ Activity\kern0.5em {Score}^{BMMN}\left({miRNA}_1\right)=\sum \limits_{k=1}^{c_1} BRCA\kern0.5em {Score}^{BMMN}\left( miRNA,\kern0.5em {miRNA}_k\right)\ast \left({c}_1/{c}_2\right) $$

$$ Activity\kern0.5em {Score}^{BMLN}\kern0.5em \left({miRNA}_1\right)=\sum \limits_{p=1}^{d_1} BRCA\kern0.5em {Score}^{BMLN}\left( miRNA,\kern0.5em {miRNA}_p\right)\ast \left({d}_1/{d}_2\right) $$

c_1_ indicates that the frequency of miRNA_1_ appears in the significantly changed miRNA-mRNA interactions. c_2_ indicates that the frequency of miRNA_1_ appears in all miRNA-mRNA interactions. d_1_ indicates that the frequency of miRNA_1_ appears in the significantly changed miRNA-lncRNA interactions. d_2_ indicates that the frequency of miRNA_1_ appears in all miRNA-lncRNA interactions.

## Results

### Breast cancer

**Fig. 4:**
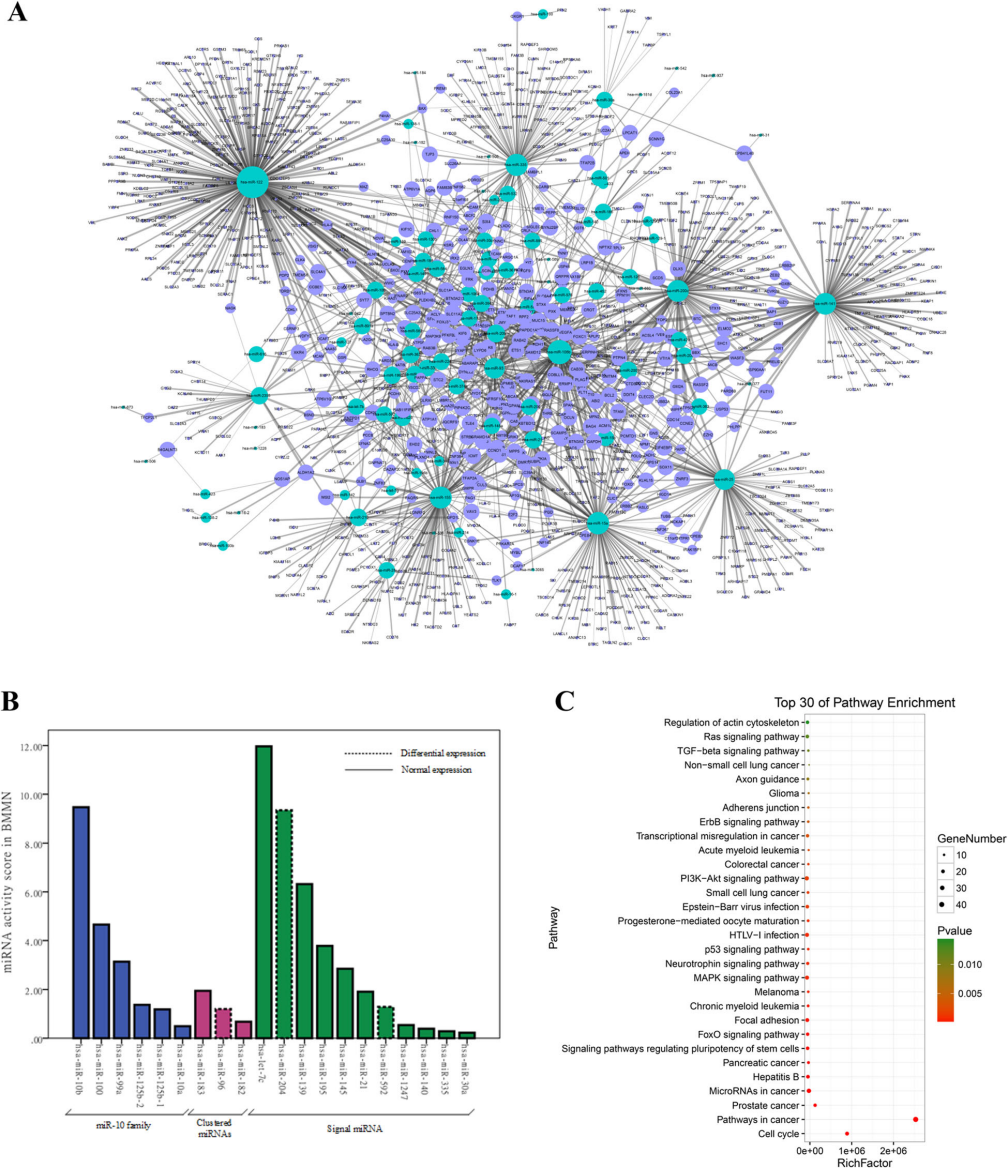
**a** The BMMN of BRCA. **b** The top 20 miRNA activity score in BMMN of BRCA. The first and second color mean the miR-10 family and the same clustered miRNA, and the third color represents signal miRNA. **c** The enrichment analyses of the 725 mRNAs in Fig. 4a

For breast cancer, we set BRCA score = 0.4 as the threshold in the BMMN. The 1078 significantly changed miRNA-mRNA interactions (Additional file 1) are selected. There are 124 miRNAs and 725 mRNAs in Fig. 4a. The blue and purple nodes represented miRNA and mRNAs, respectively. The higher the degree of the node is, the bigger the size will be. The thickness of the line reflects the BRCA score of the miRNA-mRNA interaction.

The top 20 miRNA activity scores in BMMN of BRCA are shown in Fig. 4b (The activity scores of 124 above miRNAs are show in Additional file 2). We can find that these 20 miRNAs are all related to breast cancer by searching the database HMDD. The dotted line represents the miRNAs differentially expressed. The solid line represents the miRNAs normally expressed. The first and second color mean miR-10 family and the same clustered miRNA respectively, and the third color represents signal miRNA. Many studies show that the same family and cluster of miRNAs play similar regulatory functions. The above results were in accordance with the conclusion. Moreover, the results showed many non-differentially expressed miRNAs had an important role in breast cancer.

In addition, DAVID is employed for enrichment analyses of the above 725 mRNAs. The enrichment analyses results were shown in Fig. 4c. The known cancer mechanism-related pathways are significantly enriched, such as pathways in cancer, Prostate cancer, miRNAs in cancer, Pancreatic cancer, Chronic myeloid leukemia, Melanoma, the p53 signaling pathway, small cell lung cancer, colorectal cancer, acute myeloid leukemia, Transcriptional misregulation in cancer, Glioma and Non-small cell lung cancer. These results indicate that the 725 genes were very important in cancers.

**Fig. 5:**
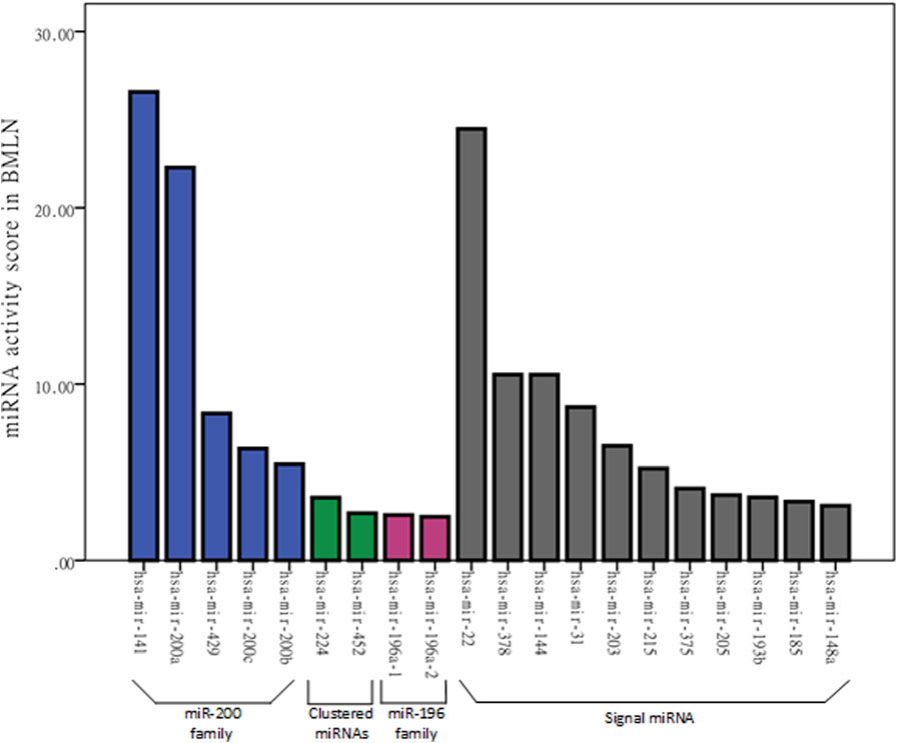
The top 20 miRNA activity scores in BMLN of BRCA. The first and third color mean the miR-200 family and miR-196 family. The second color represents the same clustered miRNA, and the fourth color represents signal miRNA

The BMLN of BRCA was also built using the abovementioned method. We set BRCA score = 0.4 as the threshold in BMLN. The 2031 significantly changed miRNA-lncRNA interactions (Additional file 3) are selected. There are 120 miRNAs and 725 lncRNAs in the above 2031 interactions. The activity scores of 120 miRNAs are show in Additional file 4). Surprisingly, all top 20 miRNA activity scores were non-differential expressed (Fig. 5). The first and third color mean the miR-200 family and miR-196 family, respectively. A previous work verified that miR-200 family is associated with breast cancer by directly targeting ADAM12-L [18]. miRNA-196 family was also reported as potent metastasis suppressors and revealed that the ratio of miR-196 family to HOXC8 mRNA is an indicator of the metastatic capability of breast tumors [19]. The second color represented the same clustered miRNA, and the fourth color represented signal miRNA. The result also showed many DM-miRNAs (non-differentially expressed miRNAs) have important role in cancers.

**Fig. 6:**
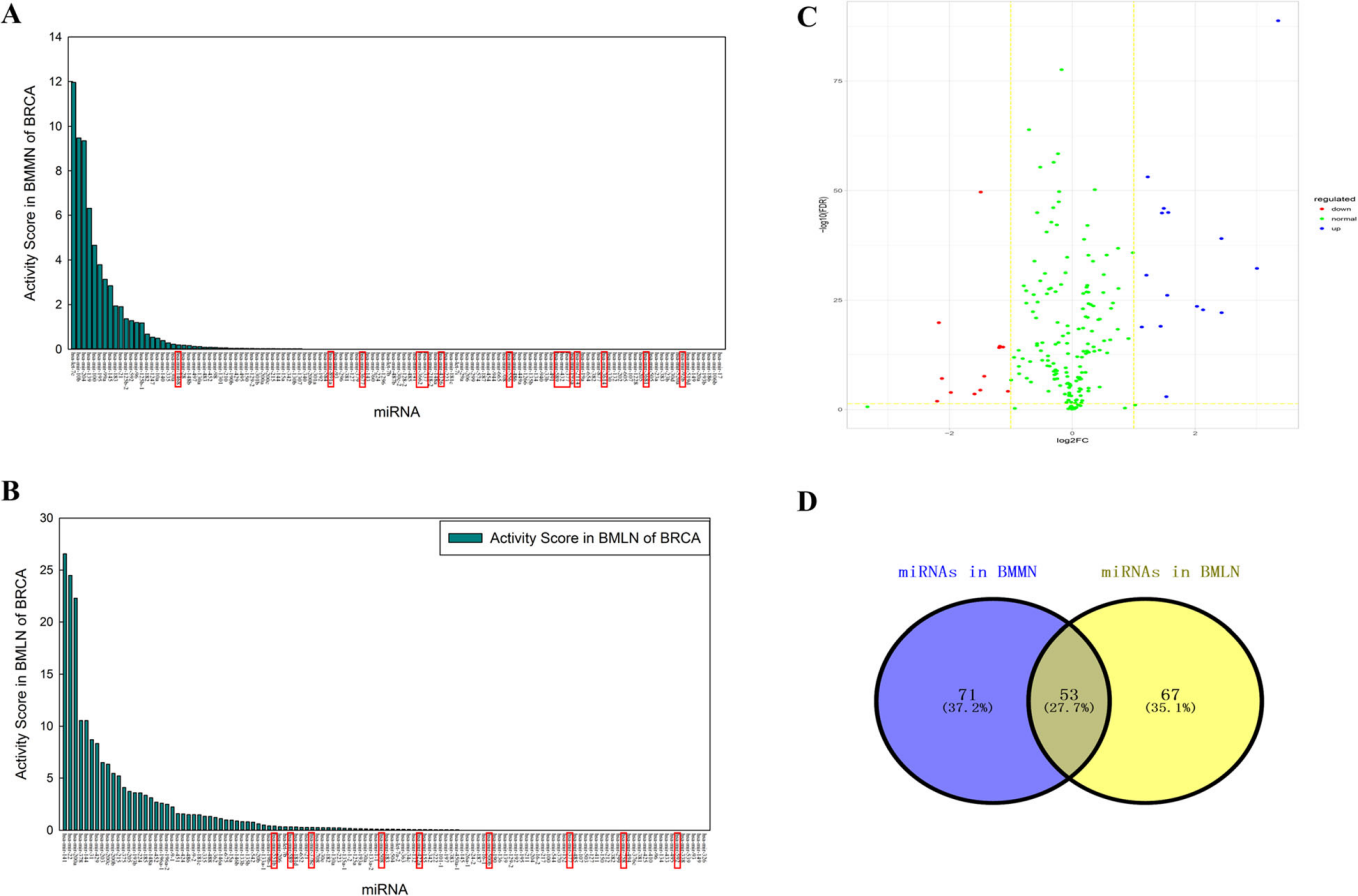
The DM-miRNAs in BMMN and BMLN. **a** The miRNA activity score in BMMN of BRCA. **b** The miRNA activity score in BMLN of BRCA. **c** The differential expression analysis of 244 miRNAs in Figure (**a** and Figure **b**). **d** The coincidence degree of two miRNA sets in Figure (**a**) and Figure (**b**)

The miRNA activity score in BMMN of BRCA were arranged in descending order (Fig. 6a). The 14 of all 124 miRNAs (miR-1468, miR-891a, miR-3677, miR-3662, miR-337, miR-4326, miR-566, miR-589, miR-432, miR-377, miR-2114, miR-3614, miR-3681, miR-92b) marked with red rectangle were not reported to be associated with BRCA, whereas other miRNAs have already been documented in BRCA. The miRNA activity score in BMLN of BRCA were arranged in descending order (Fig. 6b). The 9 of all 120 miRNAs remarked with red rectangle is not confirmed to be associated with BRCA. Figure 6c is the differential expression analysis of above 244 miRNAs. The result indicated that most of these miRNAs are non-differentially expressed. Therefore, DM-miRNAs can be discovered by calculating the activity score in BMMN and BMLN. Fig. 6d shows the coincidence degree of the above two miRNA sets (124 miRNAs in BMMN and 120 miRNAs in BMLN). The proportion of overlapping miRNA is only 27.7%. The result indicates DM-miRNAs regulating mRNA can be discovered through BMMN, and DM-miRNAs associated with lncRNA can be found through BMLN.

### The selection of machine learning algorithm

We use three types of machine learning algorithms to identify the BRCA samples and paired normal samples based on the top five changed miRNA-mRNA associations in BMMN. In general, the performance of the machine learning algorithms depends on the content of the study. Every algorithm has its own advantage. Therefore, we constructed three different classifiers using three algorithms based on the same training data set. The results show that the RF (Random Forest) algorithm outperforms the other two algorithms for classifying cancer samples and normal samples. The Area Under the Curve (AUC) measures the performance of an algorithm under different thresholds. On average, the AUC of the RF algorithm is approximately 0.9984. Compared with the AUC of the SVM (0.9909) and ANN (V-ELM) (0.9924), the RF model is robust. The sensitivity and specificity of the RF, SVM and ANN are 97.43 %  ± 0.26 and 99.53 %  ± 0.29, 96.9 %  ± 0.18 and 99.33 %  ± 0.31, 97.21 %  ± 0.22, and 99.05 %  ± 0.24 based on the 10-fold cross validation.

The acquiescent parameters C and g of the support vector machine (SVM) are 2 and 1 respectively. To improve the accuracy, the optimal parameters of SVM are 1.3471 and 0.084 per the method of the particle swarm optimization (PSO). In addition, we used an artificial neural network algorithm, called the voting based extreme learning machine (V-ELM) for comparison. ELM is a kind of quick training algorithm for generalized SLFNs [20, 21]. We selected *N* = 40 as the number of hidden layer nodes in the V-ELM model. The Random Forest (RF) method is an ensemble learning method that operates by constructing a multitude of decision trees. We chose *N* = 400 for the decision trees in RF model.

### Identification of cancer using the features of miRNAs,mRNAs, lncRNAs,miRNA-mRNA and miRNA-lncRNA interactions

**Fig. 7:**
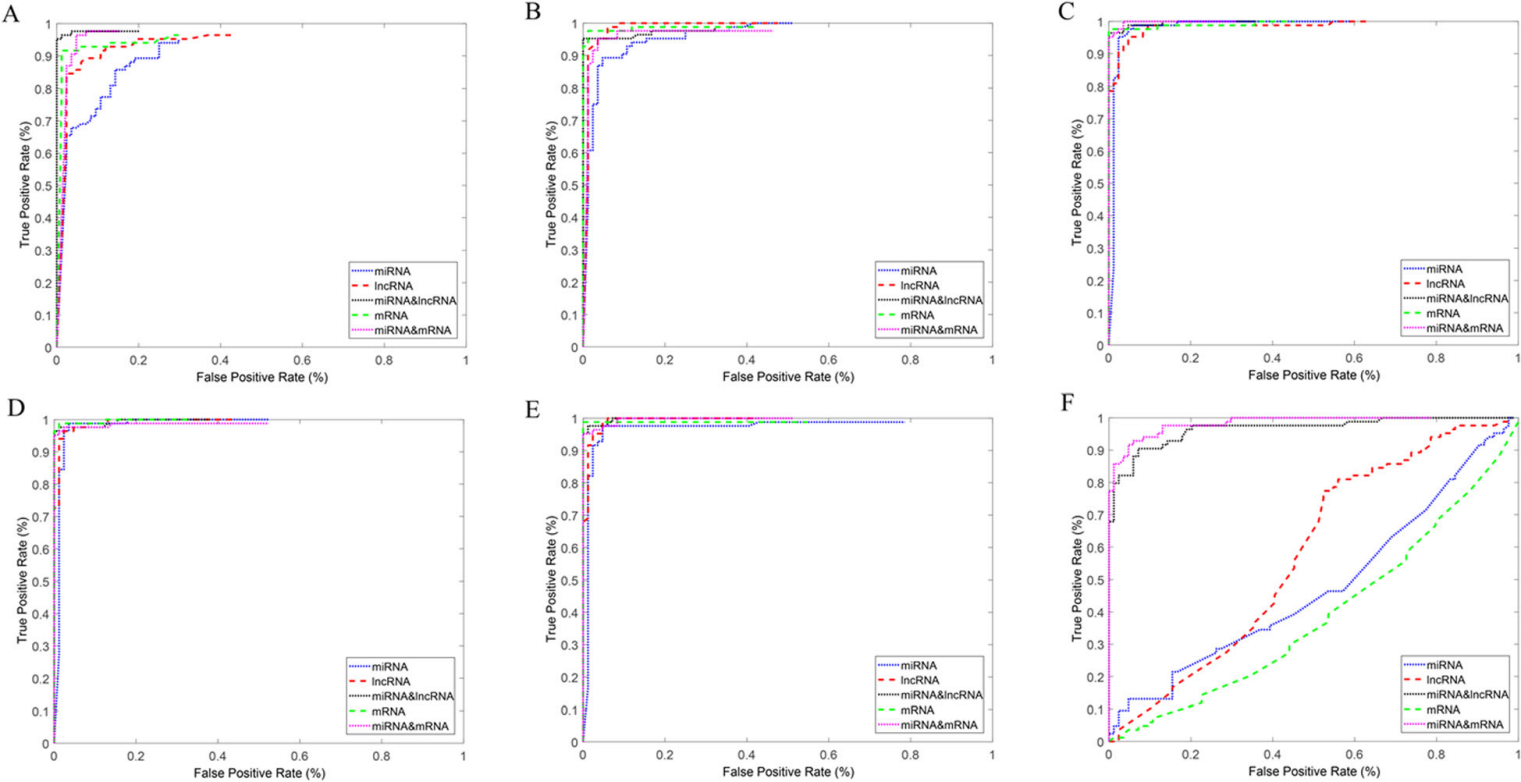
The classification performance of node biomarkers and edge biomarkers in BRCA. **a** The ROC curve based on one dimensional feature (top one differential miRNA, mRNA, lncRNA, miRNA-mRNA and miRNA-lncRNA interactions). **b-e** is the classification performance based on top two-five features (miRNA, mRNA, lncRNA, miRNA-mRNA and miRNA-lncRNA interactions). **f** The classification using the bottom five differential node biomarkers and edge biomarkers

Figure 7 shows the analysis results of RF classifier based on the top 1–5 differentially expressed mRNAs, miRNAs, lncRNAs miRNA-mRNA and miRNA-lncRNA interactions for BRCA. The results indicate that the information of miRNA-mRNA interactions is more effective. In addition, we select the bottom five differential mRNAs, miRNAs miRNA-mRNA and miRNA-lncRNA interactions for BRCA (BRCA Score > 0.4) as the features of classifier. Figure 7a is the classification performance based on one dimensional feature (top one differential miRNA, mRNA, lncRNA, miRNA-mRNA and miRNA-lncRNA interactions). The result indicated that the distinguishing ability of miRNA-mRNA and miRNA-lncRNA interactions is more effective. With the increasing of feature dimension, the performance of classification (Fig. 7b-e) based on the three node features (miRNA, mRNA, lncRNA) and two edge features (miRNA-mRNA and miRNA-lncRNA). Figure 7f is the classification result using the bottom differential node and edge features. The classification ability of edges is significantly better than that of nodes. This shows that edges contain more biological information. Therefore, the miRNA-mRNA and miRNA-lncRNA interactions can be as the effective biomarkers.

### The effectiveness of top miRNA-mRNA and miRNA-lncRNA interactions

**Fig. 8:**
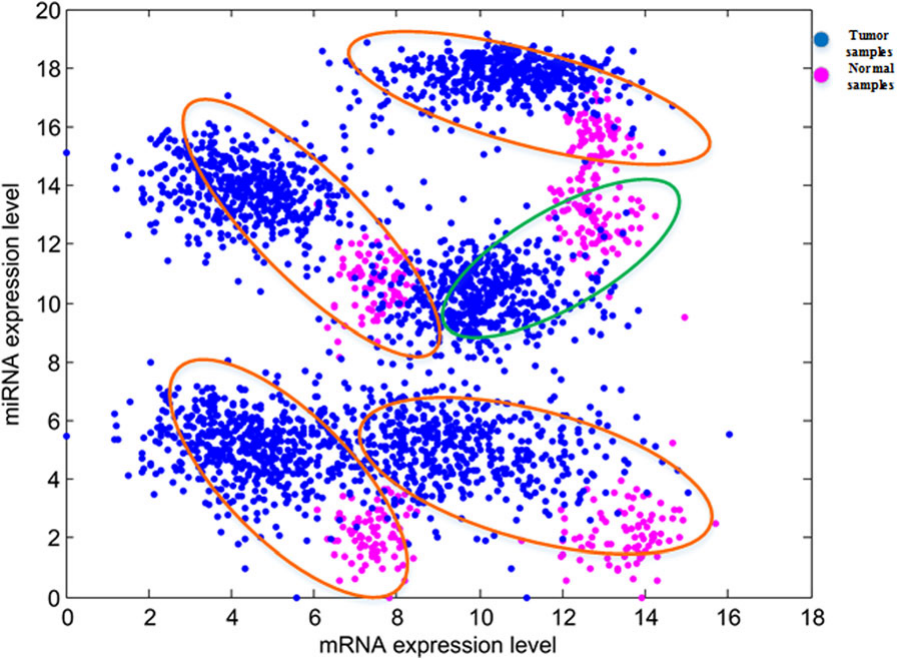
The scatter plots of the top five miRNA-mRNA interactions in BMMN of BRCA. The X axis and Y axis represent the expression level of mRNA and miRNA. The orange ellipses indicate the increase of miRNA expression results in the decrease of mRNA expression level. The miRNA-mRNA interaction in green ellipses is difficult to explain

If the difference of miRNA-mRNA and miRNA-lncRNA interactions between normal and breast cancer is more significant, the classification effect is more effective. Here, we drew scatter plots of the top 5 miRNAs with the highest BRCA score in normal and breast tumor samples. Figure 8 is the top 5 miRNA-mRNA interactions in BMMN of BRCA (miR-145-NDRG2, miR-96-HOXA5, miR-96-SYNM, miR-183- HOXA5 and miR-21-NFIB). The X axis and Y axis represent the expression level of mRNA and miRNA. The four interactions (miR-96-HOXA5, miR-96-SYNM, miR-183-HOXA5 and miR-21-NFIB) in orange ellipses indicate the increase of miRNA expression results in the decrease of mRNA expression level. The interaction (miR-145-NDRG2) in green ellipses may belong to singular interaction. What’s more, it can be seen from the scatter plot that any one of these 5 miRNA-mRNA interactions relations interactions can distinguish the normal and breast cancer samples.

**Fig. 9:**
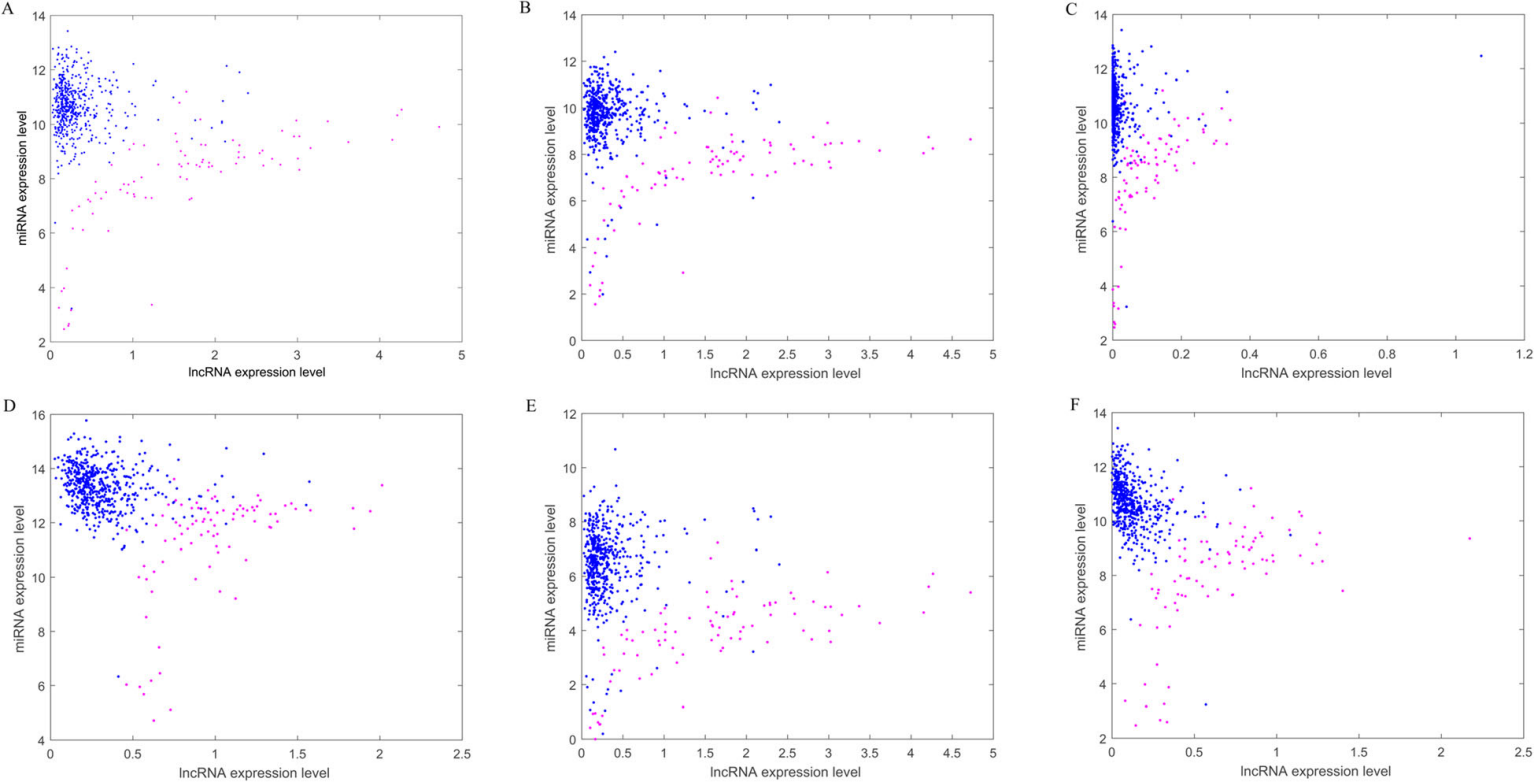
The scatter plots of the top 6 miRNA-lncRNA interactions in BMLN of BRCA. **a-f** represents the top one-six respectively. The X axis and Y axis represent the expression level of lncRNA and miRNA

Figure 9 are scatter plots of the top 6 miRNA-lncRNA interactions in BMLN of BRCA (miR-141-lnc-TRMT61B-1, miR-200a-lnc- TRMT61B-1, miR-141-lnc-PCSK9–4, miR-200c-lnc- ARL6IP5–1, miR-429-lnc-TRMT61B-1 and miR-141-GASIRR). The X axis and Y axis represent the expression level of lncRNA and miRNA. The six interactions in Fig. 9a-f indicate the increase of miRNA expression results in the decrease of mRNA expression level. Besides, any one of these 5 miRNA-lncRNA interactions relations interactions can distinguish the normal and breast cancer samples.

### The specificity of different types of cancer

**Fig. 10:**
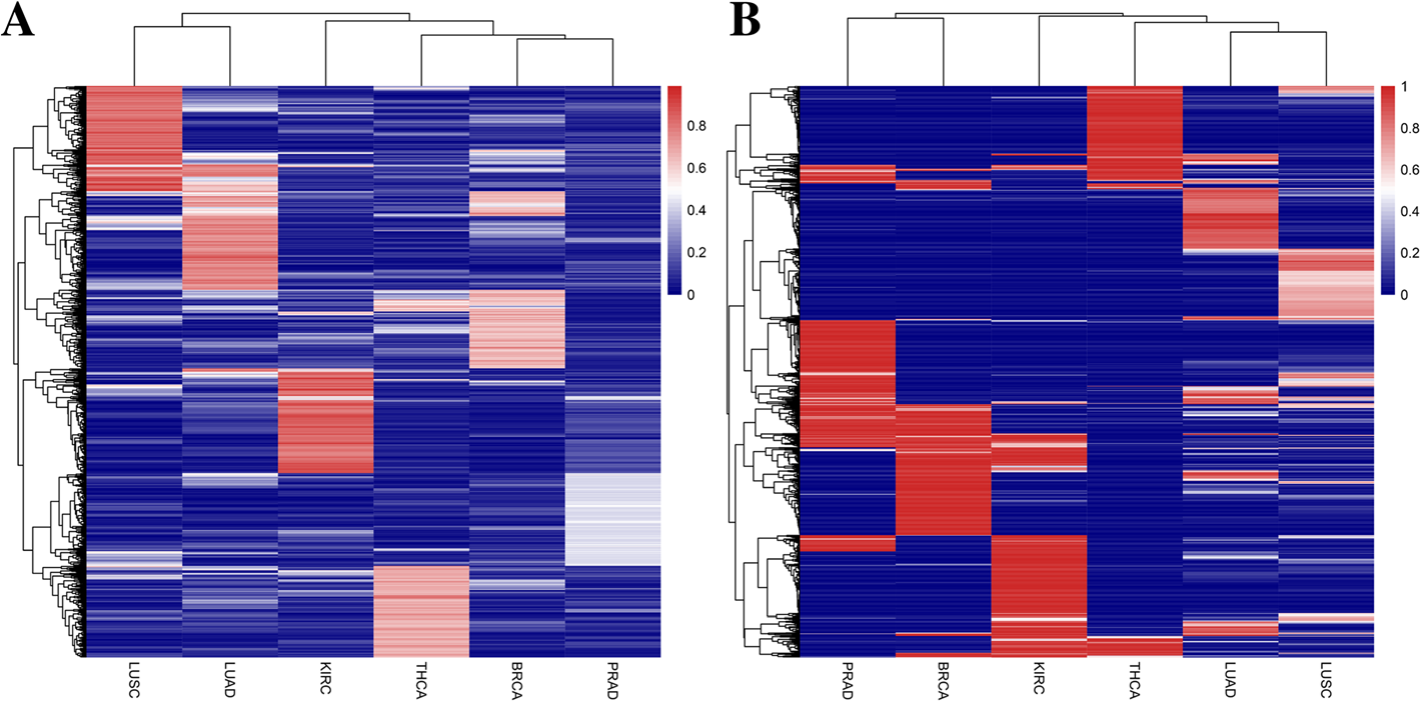
**a** and **b** represent the heat maps of the top 200 miRNA-mRNA and miRNA-lncRNA interactions in six different type of cancers

We drew the heat map by using the top 200 miRNA-mRNA and miRNA-lncRNA associations in six different type of cancers. Figure 10 is the clustering result. Six independent miRNA-mRNA and miRNA-lncRNA interaction clusters can represent six different cancers. For the two different subtypes of lung cancer (LUAD and LUSC), the difference of miRNA-mRNA and miRNA-lncRNA interactions of LUAD and LUSC is very significant. The results also indicate that the BMMN and BMLN of every kind of cancer is distinctive.

### The results of the other five cancers

1. KIRC: The Table 2 is the result of activity score of BMMN and BMLN. The ‘1’ in the third and seventh column means that miRNAs are related to kidney cancer. The ‘unknow’ is just the opposite. Fold change (FC) is used to measure differentially expressed level. We can see that most miRNAs of the Table 2 have no differential expression but are related to kidney cancer. Only two of the 24 miRNAs have not been confirmed to be associated with kidney cancer. Besides, miR-141, miR-200c and miR-200a in the first column belong to the same miRNA family. miR-192 and miR-215 in the third column belong to the same miRNA family. This result shows that the same miRNA family may play similar functions in the cancer.
2. The other four cancer: The top 10 miRNAs in BMMN and BMLN of LUSC, LUAD, THCA and PRAD are recorded in Additional file 5. We can find that 0, 2, 4 and 2 miRNAs discovered by our method are not confirmed to be associated with the above four cancers respectively.

**Table 2:** The top 12 activity score of BMMN and BMLN

miRNA	Activity in BMMN	KIRC	FC	miRNA	Activity in BMLN	KIRC	FC
miR-122	181.5	1	14.6	miR-372	17.1	1	0.22
miR-200c	60.3	1	0.48	miR-378	13.3	1	1.03
miR-141	59.3	1	0.31	miR-192	12.8	1	1.04
miR-335	26.1	1	0.74	miR-107	12.7	1	1.02
miR-199b	8.5	1	0.85	miR-217	10.5	1	0.77
miR-155	7.9	1	1.59	miR-215	9.7	1	1.36
miR-210	7.1	1	1.44	miR-216a	9.1	1	0.59
miR-200a	4.9	1	0.85	miR-203	8.7	1	0.78
miR-106b	3.5	1	1.49	miR-22	8.7	1	1.01
miR-576	3.3	Unknown	1.35	miR-216b	8.5	1	0.1
miR-25	3.2	1	1.1	miR-98	8.1	Unknown	1.01
miR-15a	3.2	1	1.24	miR-195	5.9	1	1.07

Summery, the results indicate that many DM-miRNAs can be discovered based on the BMMN and BMLN. What’s more, most high activity score miRNAs in BMMN and BMLN are non-overlapping.

## Discussion

miRNA may as the novel potential therapeutic target of cancers. However, most of miRNAs without differential expression can participate in regulation mechanism. In order to discover these DM-miRNAs, we use a novel method to discover DM-miRNAs by building a basic miRNA-mRNA network (BMMN) and miRNA-lncRNA network (BMLN). The advantage of the method is as follows.

1. The BMMN and BMLN can mining the non-differentially expressed miRNAs which play an important role in the cancer. Since most of gene is non-differentially expressed, how to find functional genes from the above genes is pivotal. The BMMN and BMLN can discover the non-differentially expressed miRNAs which play an important role in the cancer.
2. The significant difference of the BMMN and BLNM in different types of cancers. Through the clustering analysis of the top 200 miRNA-mRNA and miRNA-lncRNA interactions in six different types of cancer, we can find that the BMMNs and BMLN of six cancers are significantly different. The result indicates that the BMMN and BMLN of each cancer are very specific. It can help us distinguish the type and subtype of each cancer.
3. The edge biomarkers contain more information than the node biomarkers. The classification ability of edge biomarkers is significantly better than that of node biomarkers in BMMN and BMLN. The result shows that edges biomarkers contain more biological information.

However, our study has some limits. The miRBase database includes 2588 miRNAs, while the TCGA database only contains expression data for 1046 miRNAs. Some important miRNAs may be ignored.

## Conclusion

In brief, we proposed a new method to effectively discover DM-miRNAs by constructing BMMN and BMLN. This global miRNA-mRNA and miRNA-lncRNA interaction network will contribute to developing novel therapeutic candidates in cancers. Besides, the BMMN and BLNM may help us distinguish tumor subtypes.

## Additional files

Additional file 1: The 1078 significantly changed miRNA-mRNA interactions. (XLSX 29 kb)

Additional file 2: The activity scores of 124 above miRNAs in BMMN are shown in Additional file 2. (XLSX 13 kb)

Additional file 3: The 2031 significantly changed miRNA-lncRNA interactions. (XLSX 65 kb)

Additional file 4: The activity scores of 120 miRNAs in BMLN are shown in Additional file 4. (XLSX 13 kb)

Additional file 5: The top 10 miRNAs in BMMN and BMLN of LUSC, LUAD, THCA and PRAD are shown in Additional file 5. (ZIP 32 kb)

## Abbreviations

3’ UTRs: 3′ untranslated regions
AUC: Area Under the Curve
BMLN: BasicmiRNA-lncRNA network
BMMN: Basic miRNA-mRNA network
BRCA: Breast invasive carcinoma
CeRNA: Competing endogenous RNA
FC: Fold change
ISMLN: Individual specific miRNA-lncRNA network
ISMMN: Individual specific miRNA-mRNA network
KIRC: Kidney renal clear cell carcinoma
LUAD: Lung adenocarcinoma
LUSC: Lung squamous cell carcinoma
PCC: Pearson correlation coefficients
PMLN: Perturbed miRNA-mRNA network
PMMN: Perturbed miRNA-mRNA regulatory network
PSO: Particle swarm optimization .
RF: Random Forest (RF)
RMLN: Reference miRNA-lncRNA network
RMMN: Reference miRNA-mRNA regulatory network
SVM: Support vector machine
TCGA: The Cancer Genome Atlas
THCA: Thyroid carcinoma
V-ELM: Voting based extreme learning machine
